# Supplementary material for: Photon upconversion crystals doped bacterial cellulose composite films as recyclable photonic bioplastics
Source: Commun Mater. 2024 Sep 28;5(1):200. doi: 10.1038/s43246-024-00638-6 (PMC11438599; doi:10.1038/s43246-024-00638-6)
Supplement: Supplementary file 2 — Supplementary Information [file 43246_2024_638_MOESM2_ESM.pdf]

Photon Upconversion Crystals Doped Bacterial Cellulose Composite Films as Recyclable Photonic Bioplastics

Pankaj Bharmoria[1]*, Lukas Naimovičius[1,2], Deyaa Abol-Fotouh[1,3], Mila Miroshnichenko[1], Justas Lekavičius,[2] Gabriele De Luca[1], Umair Saeed[4], Karolis Kazlauskas,[2] Nicolas Candau,[5] Paulius Baronas,[6] Anna Roig[1], Kasper Moth-Poulsen[1,6,7,8]*

[1] Institute of Materials Science of Barcelona, ICMAB-CSIC, Bellaterra, Barcelona, 08193, Spain.

[2] Institute of Photonics and Nanotechnology, Vilnius University, Saulėtekio av. 3, LT-10257 Vilnius,Lithuania.

[3] Advanced Technology and New Materials Research Institute (ATNMRI), City of Scientific Research and Technological Applications (SRTA-City), New Borg Al-Arab, 21934, Egypt.

[4] Catalan Institute of Nanoscience and Nanotechnology (ICN2), UAB Campus, 08193 Bellaterra, Barcelona.

[5] Departament de Ciència i Enginyeria de Materials (CEM), Escola d'Enginyeria Barcelona-Est (EEBE), Universitat Politècnica de Catalunya BarcelonaTech (UPC), Av. Eduard Maristany 16, 08019 Barcelona, Spain

[6] Department of Chemical Engineering, Universitat Politècnica de Catalunya, EEBE, Eduard Maristany 10–14, 08019 Barcelona, Spain.

[7] Catalan Institution for Research & Advanced Studies, ICREA, Pg. Lluís Companys 23, Barcelona, Spain.

[8] Department of Chemistry and Chemical Engineering, Chalmers University of Technology, Kemivagen 4, Gothenburg 412 96, Sweden

*E-mails – kasper.moth-poulsen@chalmers.se; pbharmoria@icmab.es

Supporting Information

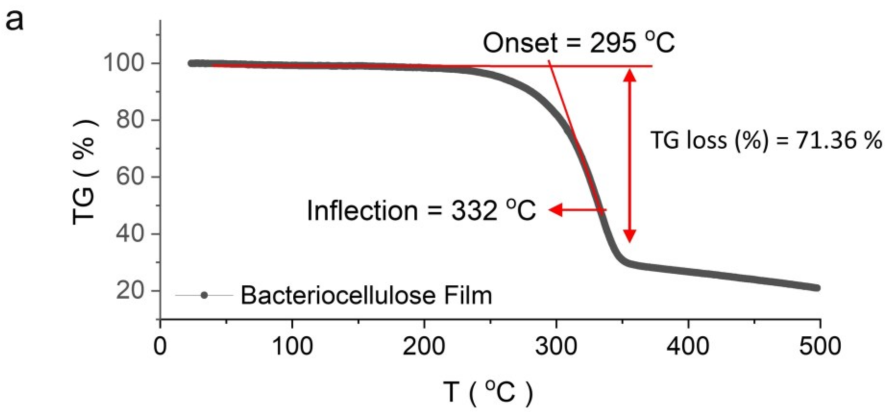

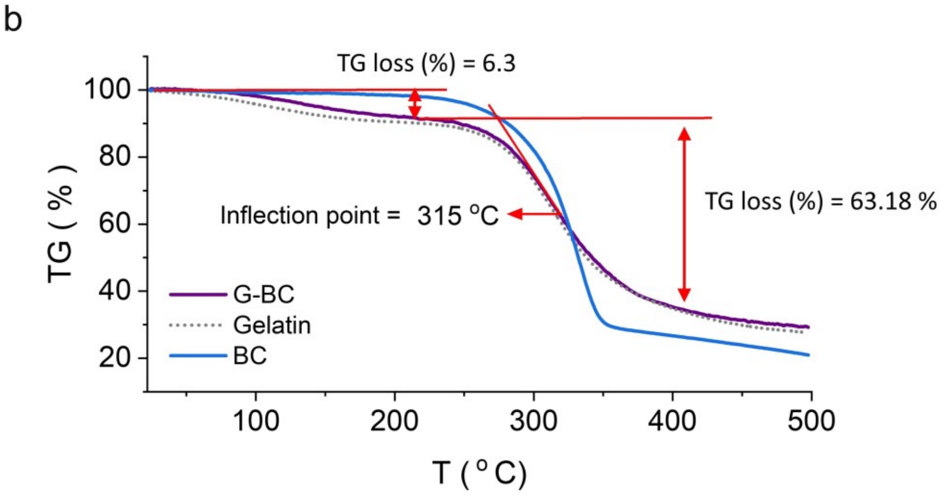

Fig. S1. Thermogravimetric measurements. TGA thermograms of **a** BC, and **b** G-BC

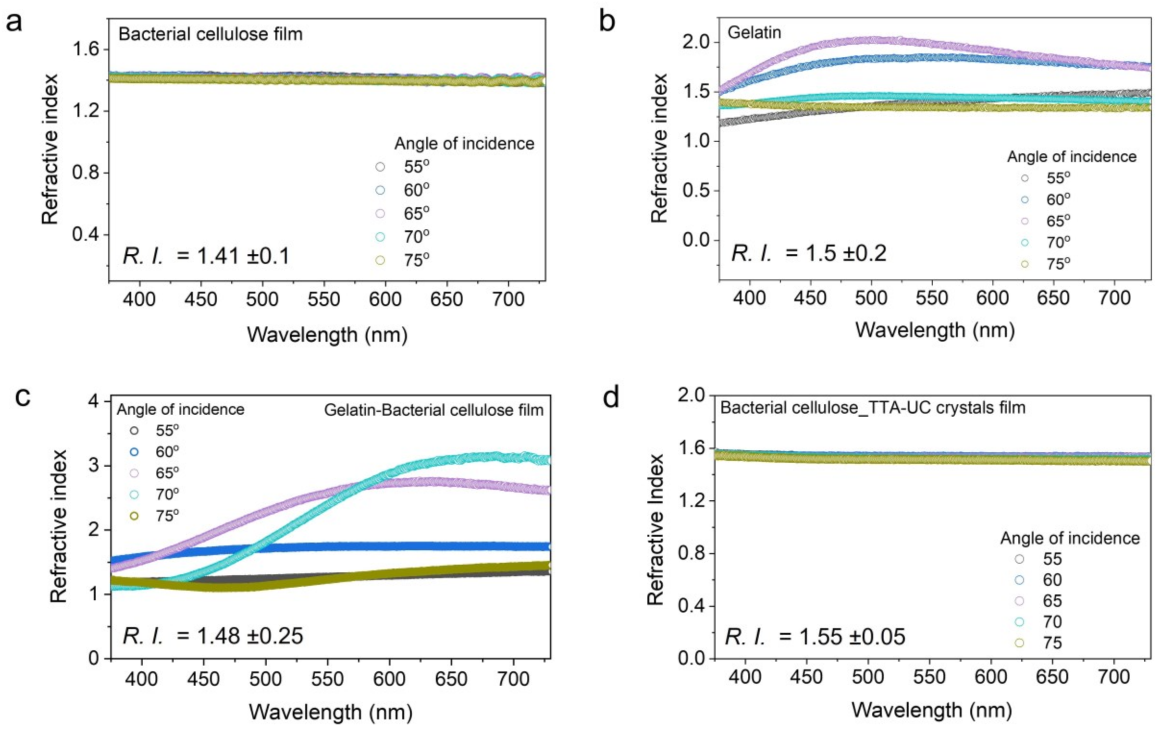

Fig. S2. Ellipsometry measurements. Refractive index vs excitation wavelength profiles at different angle of incidence of **a** bacterial cellulose, **b** gelatin, **c** gelatin-bacterial cellulose, and **d** bacterial cellulose-TTA-UC crystals.

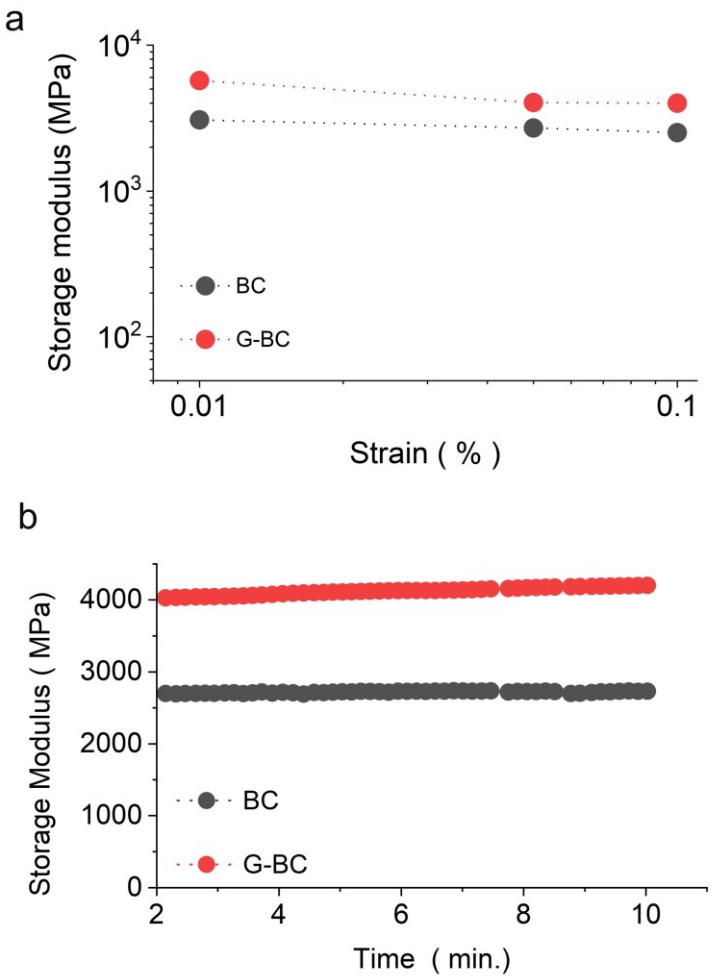

Fig. S3. Mechanical analysis. DMA profiles of BC and G-BC film. **a** Storage modulus vs strain sweep profile and **b** Storage modulus vs time sweep profile.

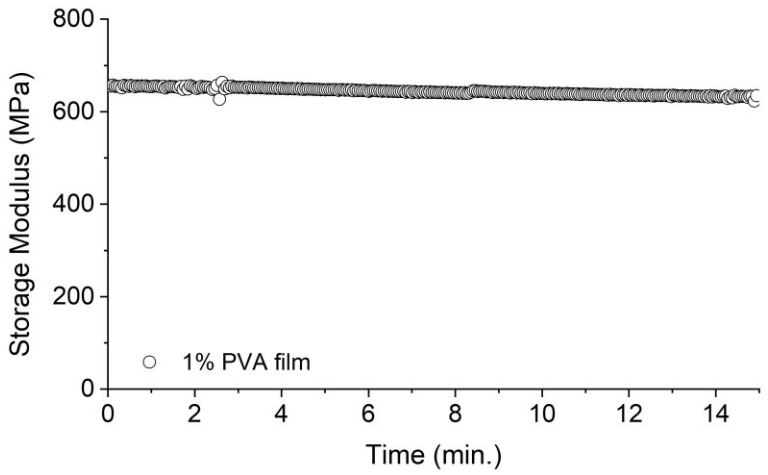

Fig. S4. Mechanical analysis. DMA profiles (Storage modulus vs time sweep profile) of 1% polyvinyl chloride film

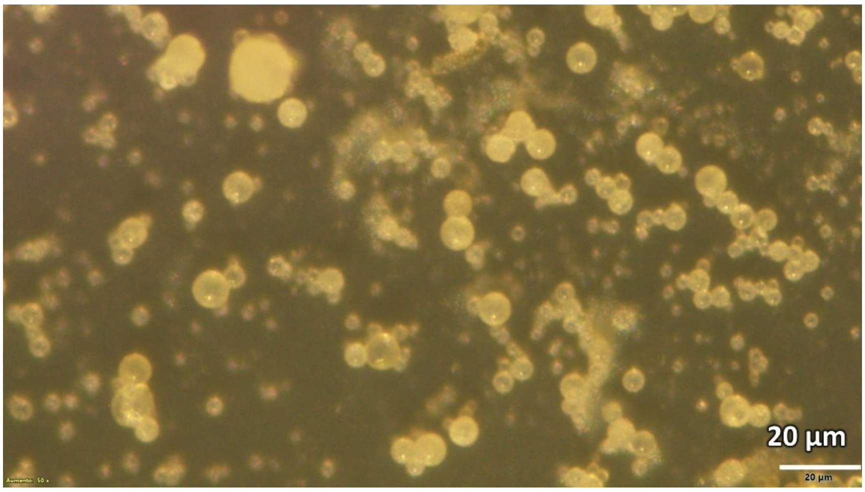

Fig. S5. Microscopy of crystals. Dark field polarized microscopic image of DPA-PdTPBP crystals doped in gelatin film.

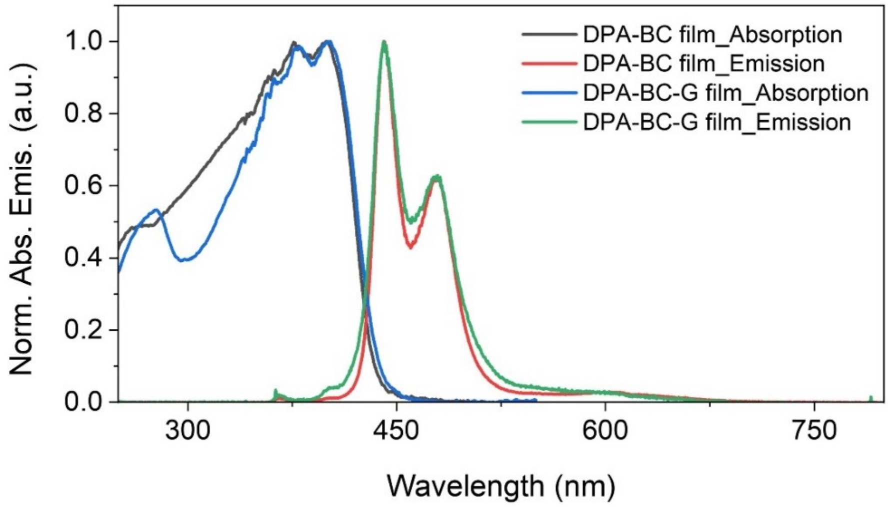

Fig. S6. Photophysical characterization of DPA. Absorption and emission spectra of DPA in BC and BC-G films.

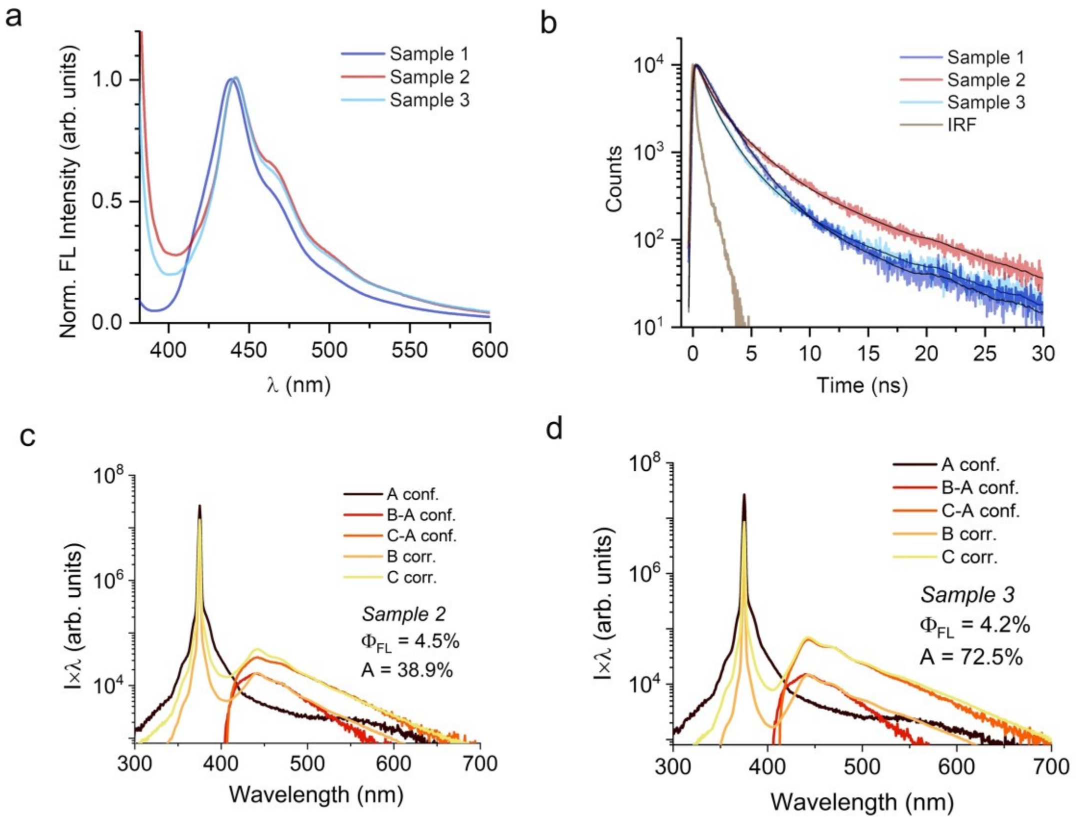

Fig. S7. Photoluminescence measurements of DPA in TTA-UC crystals. a Fluorescence emission spectrum of BC-G-DPA-PdTPBP crystals film sample 1, 2, and 3. **b** Time resolved fluorescence emission profile of BC-G-DPA-PdTPBP film sample 1, 2, and 3. **c**, **d** Emission spectra for the determination of FL quantum yield of sample 2, and sample 3 measured in the integrating sphere upon 405 nm laser excitation (A – percentage of absorbed light, Φ_{FL} – fluorescence quantum yield).

Table S1. Fluorescence lifetimes of DPA in sample 1, 2 and 3 obtained after triple exponential decay fitting. f_1, f_2 and f_3 are fractional contributions to the emission intensity of each decay time.

Sample	$f_1(\%)$	$\tau_1(ns)$	$f_2(\%)$	$\tau_2(ns)$	$f_3(\%)$	$\tau_3(ns)$
#1	29.5	0.6	57.5	1.9	13.0	7.0
#2	27.3	0.5	44.9	2.4	27.8	8.8
#3	41.6	0.5	40.6	2.0	17.8	8.2

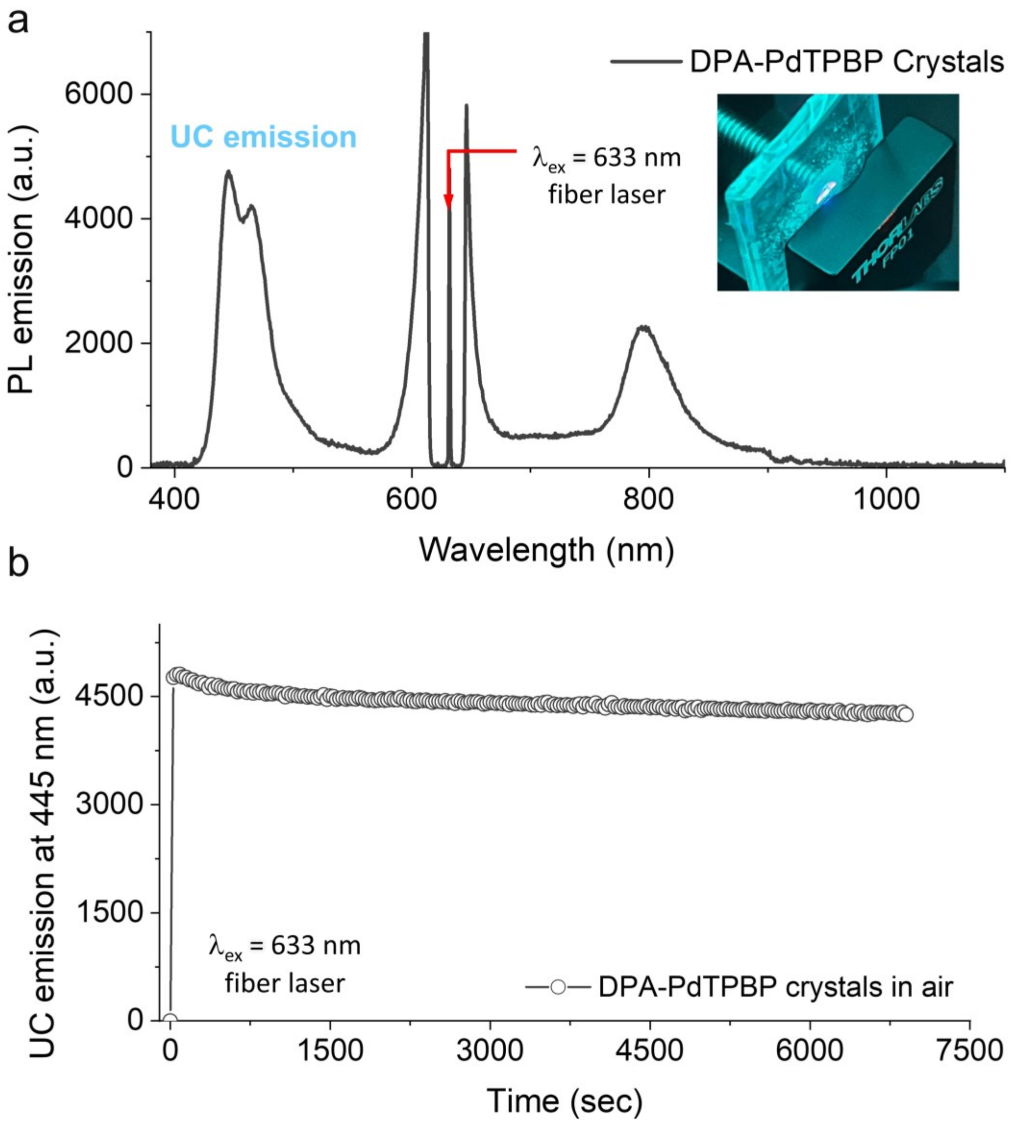

Fig. S8. Upconversion emission measurements of recycled TTA-UC crystals film. a Upconversion emission spectrum of DPA-PdTPBP crystals in air (inset showing the image of real time UC emission

upon 633 nm fiber laser excitation). **b** Time dependent UC emission of DPA-PdTPBP crystals in air (λ_{em} = 445 nm, λ_{ex} = 633 nm fiber laser).

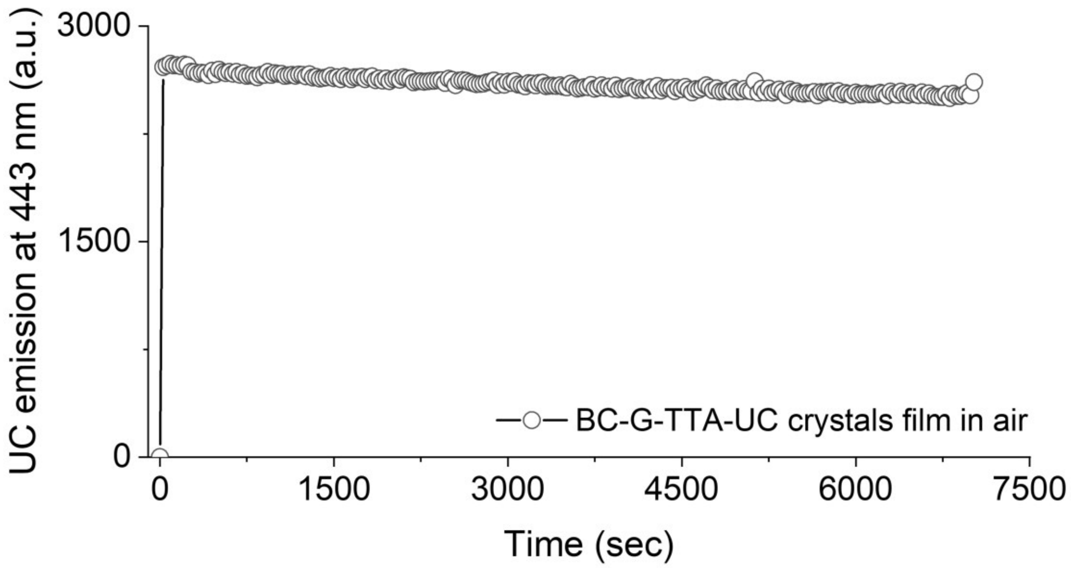

Fig. S9. Time dependent TTA-UC emission of BC-G-DPA-PdTPBP crystals in air (λ_{em} = 445 nm, λ_{ex} = 633 nm fiber laser, 10.2 W cm^{-2}).

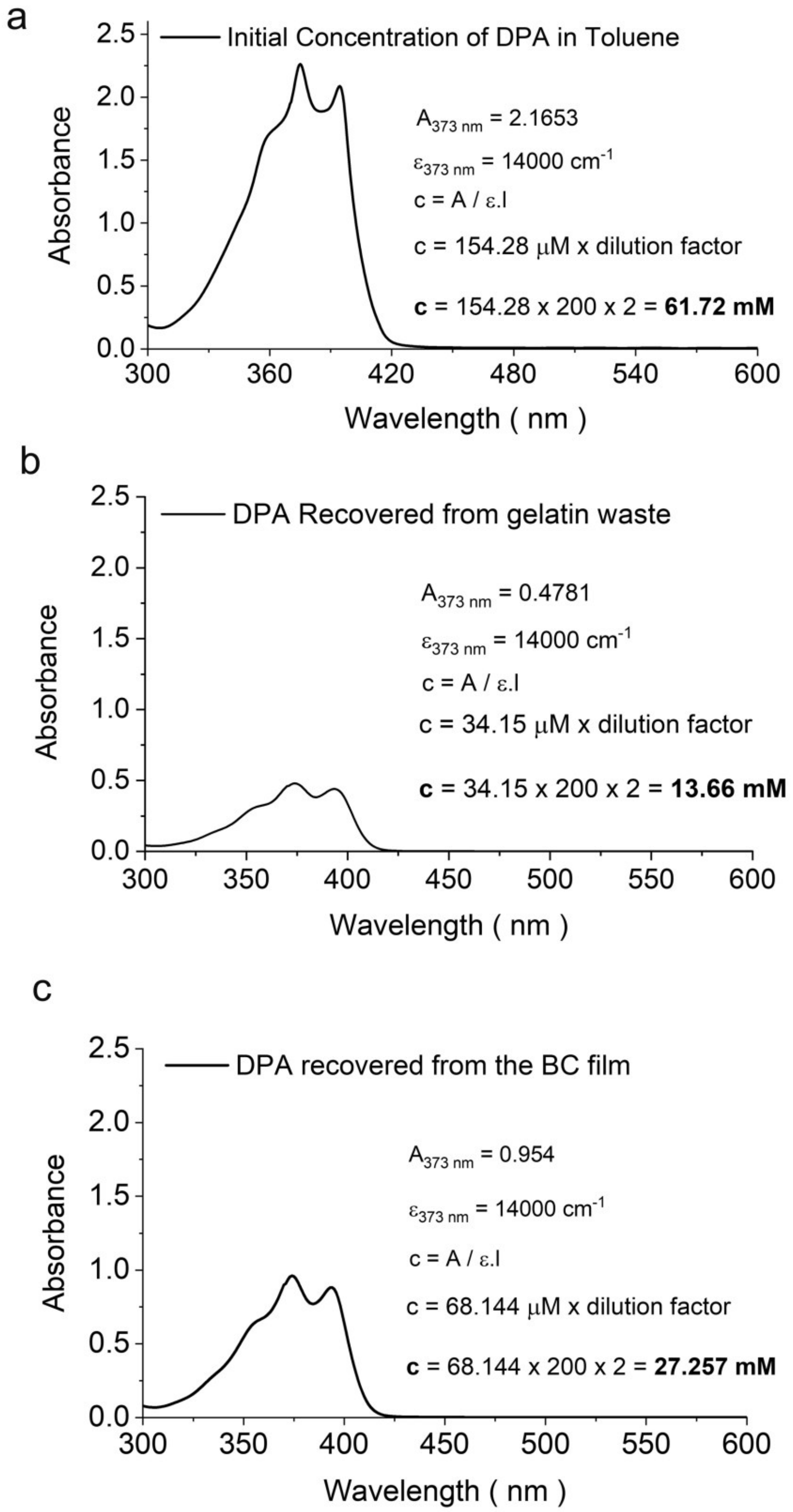

Fig. S10. Absorption measurements of the recycled DPA crystals. Absorption spectra of **a** THF solution of DPA-PdTPBP (100000:1). **b** THF solution of DPA crystals recovered from the gelatin, and **c** THF solution of DPA crystals recovered from BC film.

a

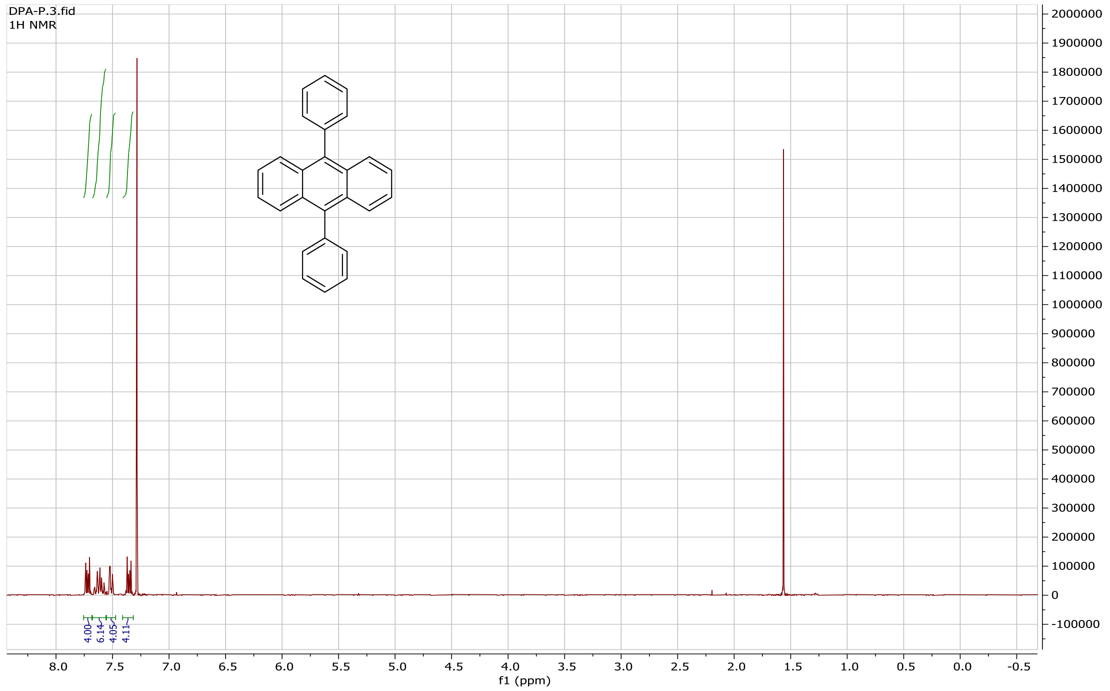

^{1}H-NMR (300 MHz, $CDCl_3$): δ 7.70 (dd, J = 6.9, 3.3 Hz, 4H), 7.62-7.54 (m, 6H), 7.48 (dd, J = 8.1, 1.8 Hz, 4H), 7.32 (dd, J = 6.9, 3.3 Hz, 4H)

b

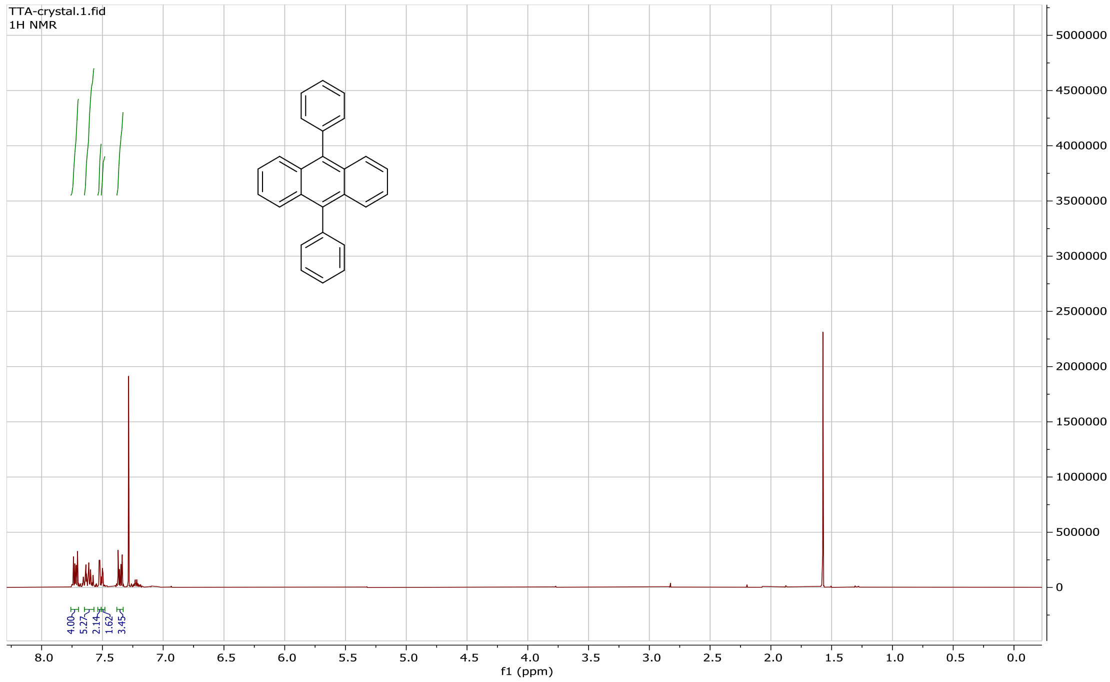

c

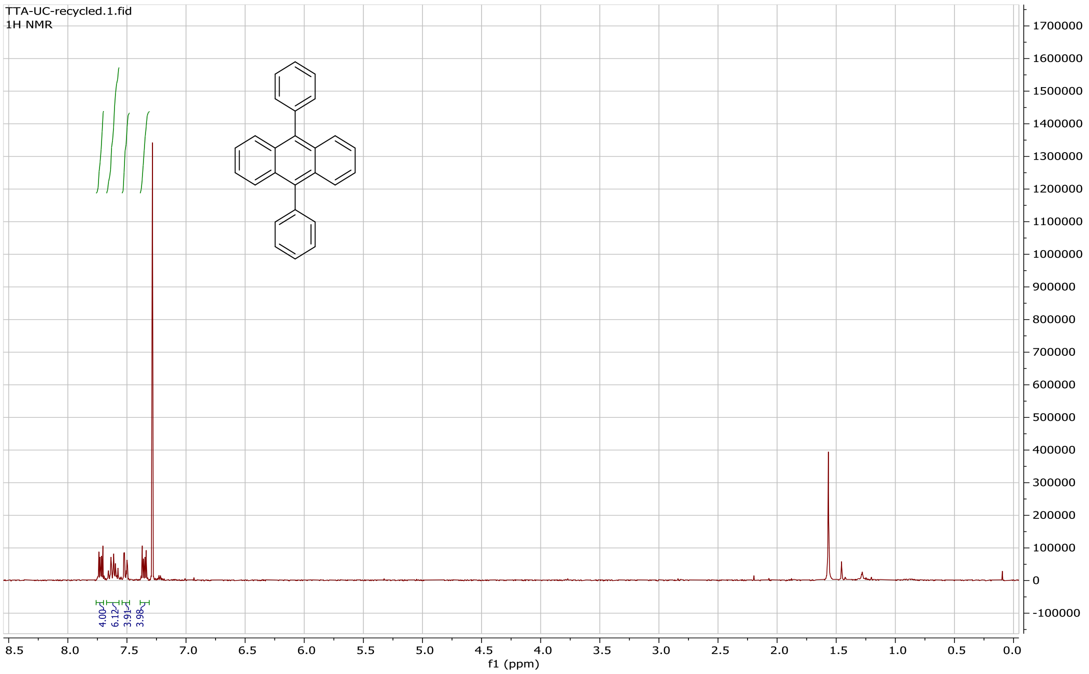

Fig. S11. Purity measurements of recycled DPA crystals. ^{1}H-NMR spectra of **a** pure DPA. **b** fresh TTA-UC crystals, and **c** recycled TTA-UC crystals.

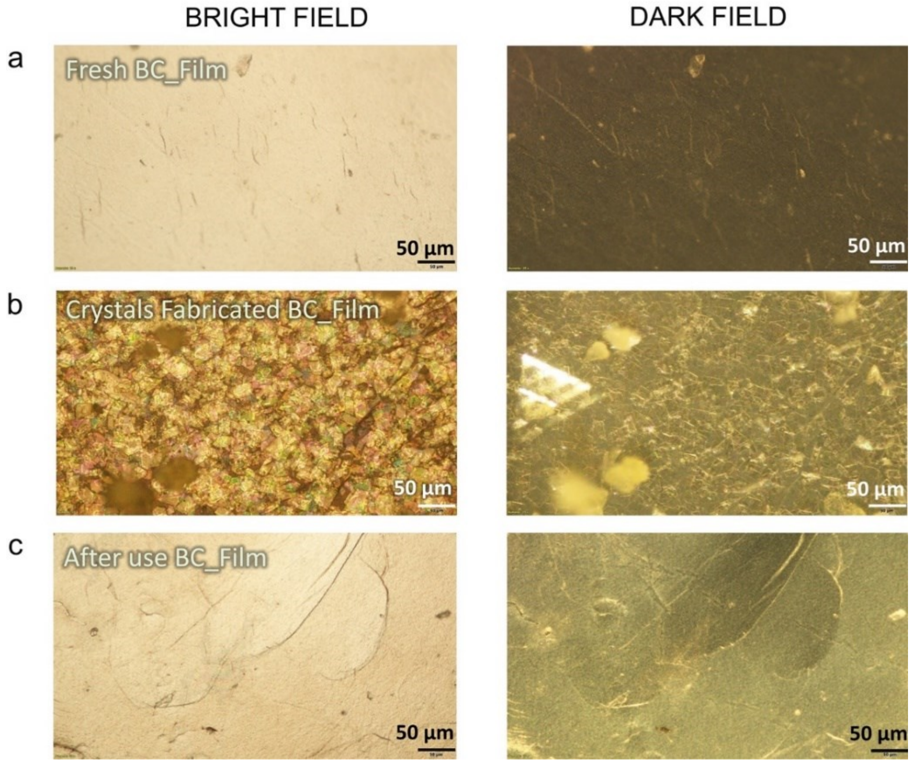

Fig. S12. Microscopy imaging of recycled materials. Bright field and dark field polarized microscopy images of **a** fresh BC film, **b** DPA-PdTPBP doped BC film, and **c** BC film after use and washing of crystals

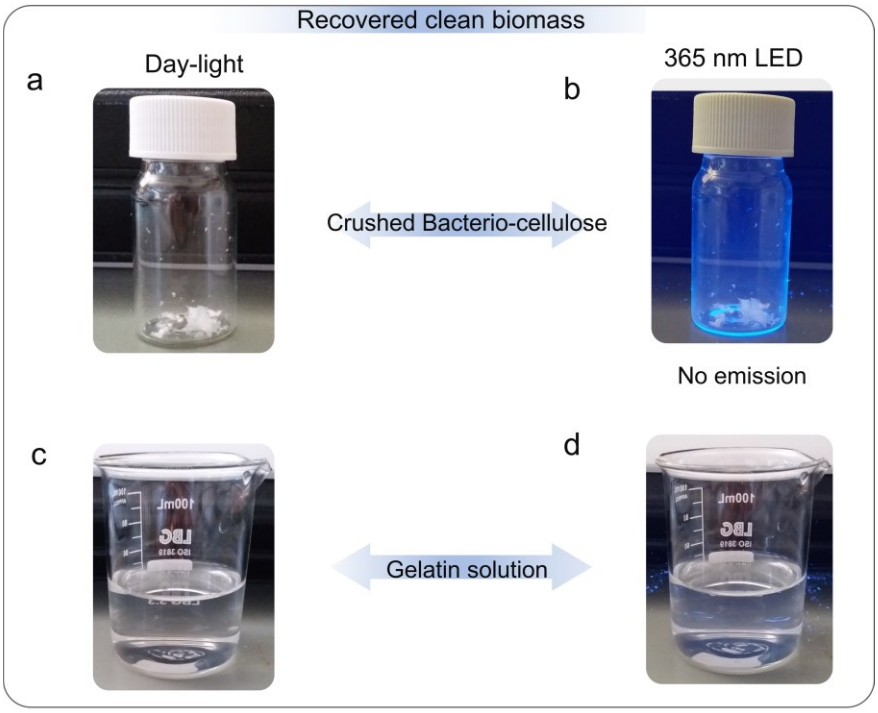

Fig. S13. Digital images of **a** and **b** recovered BC film and **c** and **d**, recovered gelatin aqueous solution in day light and UV-light of 365 nm. No emission under UV-light exposure indicates that recovered biomass is clean and does not contain any dye contaminant.

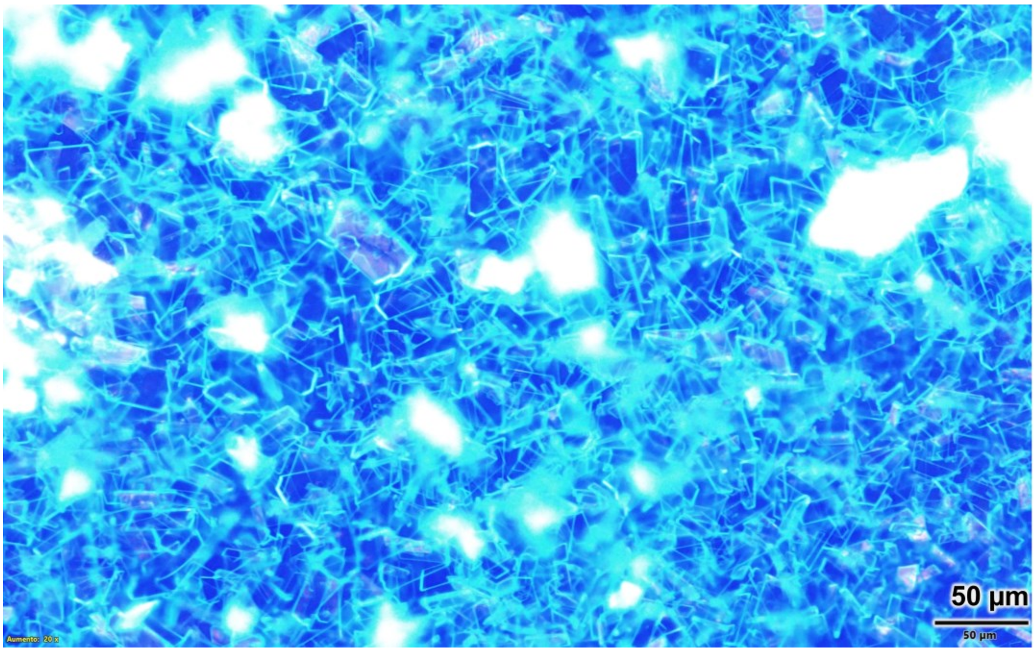

Fig. S14. Fluorescence microscopic image of the recycled BC-G-DPA-PdTPBP crystals film. (λ_{ex} = 405 nm).
